# Assessing the Role of Health Belief Model Constructs in Musculoskeletal Pains Prevention Behaviors Among Nurses in Kashan City: A Cross‐Sectional Study

**DOI:** 10.1002/hsr2.71978

**Published:** 2026-04-26

**Authors:** Esmaeil Fakharian, Mojtaba Sehat, Azam Jahangirimehr, Soudabeh Yarmohammadi

**Affiliations:** ^1^ Department of Neurosurgery Trauma Research Center, Kashan University of Medical Sciences Kashan Iran; ^2^ Department of Community Medicine, Faculty of Medicine Epidemiology, Trauma Research Center, Kashan University of Medical Sciences Kashan Iran; ^3^ Shoushtar Faculty of Medical Sciences Shoushtar Iran; ^4^ Trauma Research Center, Kashan University of Medical Sciences Kashan Iran

**Keywords:** health belief model, musculoskeletal, nurse, pain

## Abstract

**Background and Aims:**

The nurses are among vulnerable groups at risk for musculoskeletal pains (MSPs). This study aimed assessing the role of Health Belief Model (HBM) constructs in musculoskeletal pains prevention behaviors among nurses in Kashan city.

**Methods:**

A cross‐sectional analytical study was conducted from August 16, 2023, to May 4, 2024, involving 600 nurses from six hospitals in Kashan, Iran. Participants were selected through random sampling. Data were collected using a validated three‐part questionnaire covering demographics, knowledge, and HBM constructs (perceived susceptibility, perceived severity, perceived benefits, perceived barriers, self‐efficacy) related to preventive behaviors for MSPs. Statistical analysis was performed using SPSS version 21, employing descriptive statistics (mean and standard deviation) and inferential statistics (Pearson's correlation coefficient and regression). Path analysis using AMOS software explored direct and indirect effects of model constructs on the dependent variable.

**Results:**

Among the 600 nurses, 416 (69.3%) were female and 184 (30.7%) were male, with a mean age of 32.65 ± 5.10 years. Pearson correlation analyses showed significant positive correlations between all HBM constructs and preventive behaviors, except for perceived barriers. Multiple regression analyses revealed that self‐efficacy (β = 0.409, *p* < 0.001), perceived susceptibility (β = 0.241, *p* < 0.001), and knowledge (β = 0.126, *p* < 0.001) were significant predictors of preventive behaviors, collectively explaining 34.8% of the variance (R² = 0.348). Path analysis confirmed that self‐efficacy had the strongest direct effect on preventive behaviors (B = 0.411, SE = 0.042, *p* < 0.001), followed by perceived susceptibility (B = 0.241, SE = 0.067, *p* < 0.001) and knowledge (B = 0.126, SE = 0.070, *p* < 0.001).

**Conclusion:**

The HBM highlights the importance of knowledge, perceived susceptibility, and self‐efficacy in promoting preventive behaviors against MSPs among nurses. Targeted interventions to enhance these factors and ergonomic training may reduce the incidence and severity of MSPs in this population.

**Abbreviations**
CVI: content validity index
CVR: content validity ratio
C.R: critical ratio
HBM: health belief model
MSPs: musculoskeletal pains

## 1 Introduction

Jobs play an essential role in the social and economic development of any society, and working conditions can lead to many problems such as work‐related physical disorders reducing work efficiency [1]. In 2020, around 494 million people worldwide were reported to have various musculoskeletal disorders, reflecting a 123.4% increase since 1990, when approximately 221 million cases were recorded [2]. These disorders, characterized by damage or pain in various body structures such as muscles, joints, tendons, ligaments, nerves, bones, and the circulatory system [3], are widespread and recognized as significant occupational injuries in both developed and developing nations [4]. Almost a quarter of people suffer from work‐related musculoskeletal pain such as neck pain, shoulder pain, and upper limb pain, and one out of three people suffer from work‐related back pain [5].

Healthcare workers are more susceptible to MSPs than workers in construction, mining, and manufacturing industries [6]. Among healthcare staff, nurses, who play an important role in healthcare organizations, are the largest group at risk of MSPs [7]. Global epidemiological studies have shown high incidence and prevalence of back MSPs among nurses [7]. In a clinical environment, nursing staff often have to move patients, carry them to beds or other places, and lift things and do activities including excessive bending or rotation [8].

A study conducted by Rypicz et al. (2020) on nurses in Wroclaw Medical University, Poland showed that only 8% of nurses did not experience any musculoskeletal pain. In contrast, 85% of nurses had pain in more than one area of their body. The most common areas of pain included the lower back (67%), upper back (59%) and neck (66%) [9]. In a study by Koyuncu et al. (2024) conducted in Turkey, nurses reported experiencing various types of pain over the past 12 months, including lower back pain (69.5%), neck pain (68.6%), and back pain (61.9%) [10].

Effective public health initiatives designed to support health maintenance, disease risk reduction, and illness management typically necessitate behavioral adaptations across multiple tiers including individual, organizational, and community levels. Truly impactful programs in this field are consistently grounded in a deep understanding of the behaviors themselves and the environments where they take place [11, 12]. Applying health behavior theory strengthens both the design/assessment of public health initiatives and the development of research examining novel intervention approaches [12].

The selection of an appropriate theoretical framework must be guided by the specific problem, objectives, and implementation context not by a theory's popularity, novelty, or the researcher's familiarity with it [13]. The HBM stands as a seminal paradigm in health behavior theory, retaining its status as one of the most extensively recognized frameworks within the discipline. It has evolved to address contemporary domains of prevention, diagnosis, and lifestyle‐related preventive behaviors including injury mitigation [14].

According to this model, posits that health behavior adoption occurs when individuals: (1) perceive personal susceptibility to a clinically significant health threat, and (2) evaluate preventive actions as beneficial despite potential barriers. Subjective evaluations of health risks may either facilitate or impede the adoption of preventive measures [15, 16]. Higher self‐efficacy and lower perceived behavioral barriers significantly predict increased adoption of preventive measures against musculoskeletal disorders [15].

Norman and Conner's synthesis of 46 qualitative investigations established perceived severity, benefits, susceptibility, and barriers as foundational HBM constructs, with self‐efficacy subsequently incorporated as a supplementary component [17]. Alternative frameworks like the Theory of Planned Behavior which emphasizes social norms and behavioral control or Social Cognitive Theory, which prioritizes observational learning and environmental reinforcement, offer complementary insights [18].

Studies have shown that HBM is a useful framework for examining health education issues including MSPs [19, 20]. However, HBM was selected for this study due to its proven effectiveness in predicting knowledge driven and self‐efficacy‐based health behaviors in Iranian populations, and its adaptability to occupational health contexts where threat appraisal (e.g., susceptibility to MSP) is a key motivator [21, 22].

In Kashan, Iran, contextual factors may uniquely shape HBM constructs: (1) Chronic understaffing in hospitals intensifies workload pressures [23], potentially amplifying perceived barriers (e.g., “no time for posture correction”); (2) Limited access to ergonomic equipment may diminish perceived benefits of preventive actions [24], and (3) Hierarchical workplace cultures could suppress self‐efficacy if nurses feel unable to advocate for ergonomic interventions [25].

The present study offers new insights by focusing on nurses in Kashan, Iran a specific occupational context that may present unique challenges and risk factors. Distinct from earlier investigations that primarily used basic correlation or regression analyses [26, 27], this study applies path analysis to rigorously assessing the role of HBM constructs in MSPs prevention behaviors among nurses in Kashan city. Unlike traditional HBM research, which typically considers individual constructs in isolation, we incorporate knowledge not only as a direct influence on preventive actions but also as a foundational element shaping perceived susceptibility, severity, and benefits. This study aims to identify the constructs of the HBM that significantly influence preventive behaviors related to musculoskeletal disorders, thereby offering evidence‐based insights to inform the development of more effective ergonomic and educational interventions tailored specifically for healthcare workers at elevated risk of MSP.

## 2 Methods

### 2.1 Ethical Considerations

In this study, all methods were carried out in accordance with the Declaration of Helsinki and approved by the ethics committee of Kashan University of Medical Sciences, with the ethics code of IR. KAUMS. NUHEPM. REC.1402.031. All these methods followed relevant guidelines, literature review, and regulations approved by the Kashan University of Medical Sciences, Kashan, Iran. Written informed consent was obtained from all participants prior to taking part in this study.

### 2.2 Study Design, Population, Sampling Method

This cross‐sectional study of analytical type was conducted among nurses in all hospitals in Kashan city during 2023/24. Kashan has six hospitals (Shahid Beheshti, Kargar Nejad, Matini, Naqvi, Milad, and Yasrebi). Participants were selected through a stratified random sampling method. First, the total sample size (600 nurses) was proportionally allotted to each of the six hospitals based on the number of nurses employed in each hospital. Then, within each hospital, the number of nurses sampled from each department was determined proportionally according to the size of that department relative to the hospital's total nursing population. Finally, individual nurses were randomly selected from each department using a random numbers table, ensuring an unbiased and representative sample across hospitals and departments. This stratification and randomization process enhance the reproducibility and representativeness of our sample. Inclusion criteria required a minimum of 1 year of clinical experience and informed consent to participate in study. Nurses with a history of musculoskeletal disorders resulting from trauma, congenital diseases, or tumors were excluded from this study.

### 2.3 Sample Size

According to previous studies [28], the prevalence of MSPs was 98.1%. The sample size was calculated using the following formula.

N=Z2∗P∗(1−P)1−∝/2d2

Therefore, if d = Precision = (0.8), P = Expected prevalence or proportion = (98.1) and Z = Z statistic for a level of confidence (1.96 for 95% confidence level), the sample size was considered 518 people. Taking into account the drop‐out rate, the sample size was considered 600 people.

### 2.4 Data Collection

The data collection method was implemented as follows: After coordinating with hospitals authorities in Kashan, sampling was conducted. A trained interviewer collected the data by visiting the hospital wards during morning and evening shifts. The interviewer explained the purpose of the project and ensured the confidentiality of the participants' information before distributing the questionnaires to the nurses individually. The completed questionnaires were then collected after the nurses finished them. The questionnaire completion took about 15 min.

### 2.5 Measurement Tool

The data collection tool consisted of a three‐part questionnaire. The first section included eight questions regarding the personal and occupational information of nurses, categorized qualitatively (gender, age, level of education, economic status, marital status, job experience, working hours per week and experience of pain since last week). The second part of the questionnaire was related to the questions of knowledge, which was made by the researchers by reviewing the literature. This section had eight questions in the field of risk factors that increase MSPs, as well as information that reduces MSPs and these questions are classified quantitatively. A correct answer had two points, a wrong answer had one point, and an ‘I don't know’ answer had zero point. The knowledge score was in the range of 16 to zero. Consequently, the minimum score was zero, while the maximum score was 16.

The third section of the questionnaire included questions related to the HBM constructs (perceived susceptibility, perceived severity, perceived benefits, perceived barriers, self‐efficacy and behavior). These questions were developed by the researchers through a review of the literature, and those concerning model structures were also categorized quantitatively. The construct of perceived susceptibility included five questions based on a 5‐point Likert scale from 5 (completely agree) to 1 (completely disagree). The obtainable score of this construct was in the range of 25 to 5. An example of perceived susceptibility question is “Prolonged standing (over 20 min) can lead to pain in my back, neck, and shoulders”. Perceived severity construct included five questions based on a 5‐point Likert scale from 5 (completely agree) to 1 (completely disagree). The obtainable score of this construct was in the range of 25 to 5. An example of perceived severity question is “I am unable to focus on my work due to the pain in my back, neck, and shoulders”.

The construct of perceived benefits consisted of four questions based on a 5‐point Likert scale ranging from 5 (completely agree) to 1 (completely disagree). The obtainable score of this construct was in the range of 20 to 4. An example of perceived benefits question is “By maintaining proper sitting and walking posture, I can avoid the costs of treatment for my back, shoulders, and neck”. The construct of perceived barriers included four questions based on a 5‐piont Likert scale from 5 (completely disagree) to 1 (completely agree). Scoring of the perceived barriers construct was done in reverse sound. The obtainable score of this construct was in the range of 20 to 4. An example of perceived barriers question is “Working long hours is one of the causes of my musculoskeletal pain”.

The self‐efficacy construct had six questions based on a 5‐point Likert scale from 5 (completely agree) to 1 (completely disagree). The obtainable score of this construct was in the range of 30 to 6. An example of self‐efficacy construct question is “I can use stretching and strengthening exercises to prevent fatigue”. Finally, there were eight questions related to preventive behavior of MSPs. The Likert scale included 5 (always), 4 (sometimes), 3 (often), 2 (rarely), and 1 (never). The acquisition score for this structure is 8 to 40. An example of behavior question is “I take short breaks and change my body position during the shift”.

### 2.6 The Questionnaire Validity and Reliability

To create the questionnaire, initial bibliographic research was conducted on the topic [29, 30, 31]. Face‐to‐face interviews were held with 10 nurses (who did not participate in subsequent phases of the study) to assess the qualitative face validity. They were asked about the difficulty level, appropriateness, and clarity of the questionnaire items. To evaluate the quantitative content validity of the tool, 10 experts (health education, orthopedist, and nurses) were consulted regarding the necessity, relevance, simplicity, and clarity of each question. Based on Lawshe's criteria [32], for a panel of 10 experts, items with a content validity ratio (CVR) exceeding 0.62 are deemed statistically significant. In the present study, the calculated CVR of 0.79 meets this threshold, confirming the essentiality of the included items. For the content validity index (CVI), we applied the method proposed by Waltz and Bausell [33], where scores below 0.70 are considered unacceptable. Our obtained CVI of 0.8 surpasses this cutoff, demonstrating adequate content validity for the overall questionnaire.

Cronbach's alpha values were determined for knowledge (0.803), perceived severity (0.997), perceived susceptibility (0.990), perceived barriers (0.885), perceived benefits (0.881), self‐efficacy (0.862), and preventive behavior (0.888). The reliability of the questionnaire was assessed using a test‐retest method. In this process, 30 nurses completed the questionnaire twice, with a 2‐week interval, and the scores from both stages were compared. The reliability coefficient was found to be 0.9.

### 2.7 Data Analysis

Quantitative variables were summarized using descriptive statistics, including mean and standard deviation (SD), while qualitative variables were described by frequencies and percentages (%). Pearson's correlation coefficient (r) was calculated to evaluate the strength and direction of linear associations among the constructs of the HBM.

Multiple linear regression analyses were conducted as pre‐specified analyses to assess the relationships between HBM constructs, knowledge, and preventive behaviors related to MSPs. Subsequently, confirmatory path analysis was performed to investigate both the direct and indirect effects of these constructs on preventive behaviors. Prior to conducting regression and path analyses, key statistical assumptions were rigorously evaluated to ensure valid inferences: Normality of continuous variables was tested using the Shapiro‐Wilk test. Homogeneity of variances was assessed by Levene's test. Multicollinearity was evaluated using variance inflation factors (VIF), with values below 5 indicating no concerning multicollinearity. All assumptions were satisfactorily met, supporting the appropriateness of the applied methods.

Model fit for path analysis was assessed using a suite of indices, including Chi‐square statistic (*χ*²), degrees of freedom (df), relative Chi‐square (*χ*²/df), Tucker‐Lewis Index (TLI), Comparative Fit Index (CFI), Normed Fit Index (NFI), Incremental Fit Index (IFI), Root Mean Square Error of Approximation (RMSEA), and Root Mean Square Residual (RMR), following established psychometric standards.

No significant missing data occurred in the dataset; thus, all analyses were conducted on complete cases. All hypothesis tests were two‐sided, and the a priori threshold for statistical significance was set at *p* < 0.05. Statistical data management and preliminary analyses were performed using IBM SPSS Statistics software, version 21 (IBM Corp., Armonk, NY, USA). Path analysis modeling was carried out in IBM AMOS software, version 21 (IBM Corp., Armonk, NY, USA).

This statistical approach follows recommendations of the SAMPL (Statistical Analyses and Methods in the Published Literature) guidelines to ensure transparency, reproducibility, and clarity in reporting statistical methods [34].

## 3 Results

In this study, out of 600 nurses participating in the study, 416 (69.3%) were female and 184 (30.7%) were male. The mean and standard deviation of the age of the nurses was 32.65 ± 5.1 years, 445 subjects (74.2%) were married, 438 subjects (73%) had a bachelor's degree, the average income was 150 ± 22 million Rials, the mean and standard deviation of nurses' work experience was 8.58 ± 3.3 years and out of the 600 nurses surveyed, 497 (82.8%) reported experiencing MSPs. Other demographic information is shown in Table 1.

**Table 1:** Demographic characteristics of research participants (*N* = 600).

Characteristics	Categories	*N* (%)
Gender	Female	416 (69.3)
	Male	184 (30.7)
Age (year)	20–30	195 (32.5)
	30–40	219 (36.5)
	40–50	151 (25.2)
	50–60	35 (5.8)
	Mean ± SD	32.65 ± 5.1
Level of education	Diploma and associate degree	102 (17.0)
	Bachelor degree	438 (73.0)
	Master degree	60 (10.0)
Economic status	High	20 (3.3)
	Average	467 (77.8)
	Low	113 (18.8)
Marital status	Single	143 (23.8)
	Married	445 (74.2)
	Widow/divorced	12 (2.0)
Job experience (year)	1–5	204 (34.0)
	6–10	141 (23.5)
	11–15	94 (15.7)
	16–20	94 (15.7)
	21–25	39 (6.5)
	26–30	23 (3.8)
	31–35	5 (0.8)
	Mean ± SD	8.58 ± 3.3
Hours worked weekly	25 to 50 h	347 (57.8)
	Over 50 h	253 (42.2)
Experience of pain (since last week)	Yes	497 (82.8)
	No	103 (17.2)

Abbreviation: MSPs, musculoskeletal pains.

The mean and standard deviation of knowledge and HBM constructs are shown in Table 2. The highest mean and standard deviation were related to preventive behaviors (24.97 ± 5.61), knowledge (22.32 ± 2.79), self‐efficacy (19.14 ± 4.73), and perceived susceptibility (19.07 ± 2.96).

**Table 2:** The mean and standard deviation of the health belief model dimension scores and MSPs preventive behaviors in the studied nurses (*N* = 600).

Variable	Mean ± SD
Knowledge	22.32 ± 2.79
Perceived susceptibility	19.07 ± 2.96
Perceived severity	17.59 ± 4.07
Perceived benefits	16.16 ± 2.36
Perceived barriers	11.74 ± 3.14
Self‐efficacy	19.14 ± 4.73
Preventive behaviors	24.97 ± 5.61

Abbreviation: MSPs, musculoskeletal pains.

Table 3 presents the correlation matrix for HBM constructs with preventive behaviors related to MSPs. The results indicate a direct and statistically significant positive correlation between perceived susceptibility, perceived severity, perceived benefits, and self‐efficacy with the preventive behavior regarding MSPs. In contrast, perceived barriers exhibited an inverse (negative) correlation with preventive behaviors related to MSPs. So, the strength of the positive association's ranges from moderate to strong, with self‐efficacy showing the strongest positive correlation (r = 0.474, *p* < 0.001).

**Table 3:** Pearson correlation coefficient of the HBM constructs and MSPs preventive behaviors in the studied nurses (*N* = 600).

Variables	Perceived susceptibility	Perceived severity	Perceived benefits	Perceived barriers	Self‐efficacy	Preventive behaviors
Perceived susceptibility	1	—	—	—	—	—
Perceived severity	R = 0.371** *p* = < 0.001	1	—	—	—	—
Perceived benefits	R = 0.367** *p* = < 0.001	R = 0.247** *p* = < 0.001	1	—	—	—
Perceived barriers	R = −0.157** *p* = < 0.001	R = −0.288** *p* = < 0.001	R = −0.157** *p* = < 0.001	1	—	—
Self‐efficacy	R = 0.221** *p* = < 0.001	R = 0.164** *p* = < 0.001	R = 0.182** *p* = < 0.001	R = 0.046 *p* = 0.265	1	—
Preventive behaviors	R = 0.350** *p* = < 0.001	R = 0.215** *p* = < 0.001	R = 0.137** *p* = < 0.001	R = −0.026 *p* = 0.532	R = 0.474** *p* = < 0.001	1

*Note:* Correlation is significant at the 0.01 level (2‐tailed).

Abbreviations: HBM, Health Belief Model; MSPs, Musculoskeletal pains.

Table 4 presents regression coefficients predicting preventive behaviors. Self‐efficacy was the strongest predictor (β = 0.409, SE = 0.035, t = 11.65, *p* < 0.001). Perceived susceptibility (β = 0.241, SE = 0.042, t = 6.82, *p* < 0.001) and knowledge (β = 0.126, SE = 0.035, t = 3.63, *p* < 0.001) also significantly predicted behaviors. The model explained 34.8% of variance (R² = 0.348).

**Table 4:** Factors predicting the preventive behaviors of subjects based on HBM constructs.

Coefficients						
Model	Unstandardized coefficients		Standardized coefficients	*t*	Sig.	R square
	B	Std. error	Beta			
(Constant)	1.326	1.904	—	0.696	0.486	0.348
Self‐efficacy	0.486	0.042	0.409	11.652	< 0.001	
Perceived susceptibility	0.457	0.067	0.241	6.816	< 0.001	
knowledge	0.252	0.070	0.126	3.626	< 0.001	
a. Dependent variable: behavior						

Abbreviation: HBM, health belief model.

### 3.1 Path Analysis

**Figure 1:**
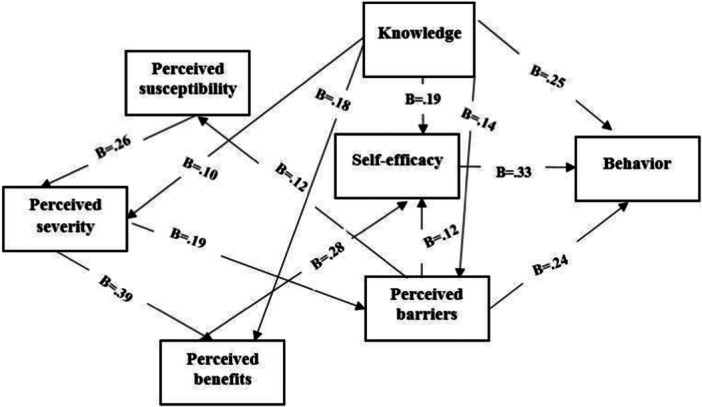
Relationship model between Health Belief Model constructs and musculoskeletal pains preventive behaviors in 600 nurses.

As shown in Figure 1 and Table 5, self‐efficacy exerted the largest direct effect on preventive behavior (B = 0.411, SE = 0.042, *p* < 0.001), followed by perceived susceptibility (B = 0.241, SE = 0.067, *p* < 0.001) and knowledge (B = 0.126, SE = 0.070, *p* < 0.001). The model confirmed indirect effects of perceived barriers, benefits, and severity on preventive behaviors through self‐efficacy and perceived susceptibility. the model fit indices also confirmed the appropriateness of the model: Chi‐square=12.10, degree of freedom = 8, Chi‐squared statistic divided by degree of freedom= 1.51, Tucker‐Lewis index= 0.978, Normed Fit Index:0.978, Root Mean Square Residual= 0.082, Comparative Fit Index= 0.992, Incremental Fit Index:0.992 and Root Mean Square Error of Approximation=0.029.

**Table 5:** Exploring direct and indirect effects of HBM constructs on MSPs preventive behaviors and Path coefficients and the variance in HBM constructs in nurses.

Variable		Path	B	S. E	C.R	*p*
**Perceived susceptibility**		Knowledge → perceived susceptibility	0.146	0.043	3.612	< 0.001
Direct effects	0.241					
Indirect effects	0.091	Perceived susceptibility → perceived severity	0.372	0.052	9.807	< 0.001
Total effects	0.332					
**Knowledge**						
Direct effects	0.126	Knowledge → perceived benefits	0.132	0.032	3.505	< 0.001
Indirect effects	0.055					
Total effects	0.181	Perceived susceptibility → perceived benefits	0.317	0.032	7.836	< 0.001
**Perceived severity**						
Direct effects	< 0.001	Perceived severity → perceived barriers	−0.288	0.030	−7.359	< 0.001
Indirect effects	0.038					
Total effects	0.038	Perceived severity → perceived benefits	0.114	0.023	2.851	0.004
**Perceived benefits**						
Direct effects	< 0.001	Perceived benefits → self‐efficacy	0.117	0.085	2.751	0.006
Indirect effects	0.048					
Total effects	0.048	Perceived barriers → self‐efficacy	0.124	0.062	3.023	0.003
**Perceived barriers**						
Direct effects	< 0.001	Perceived severity → self‐efficacy	0.114	0.051	2.614	0.009
Indirect effects	0.051					
Total effects	0.051	Perceived susceptibility → self‐efficacy	0.149	0.071	3.353	< 0.001
**Self‐efficacy**						
Direct effects	0.411	Self‐efficacy → preventive behavior	0.411	0.042	11.681	< 0.001
Indirect effects	< 0.001	Perceived susceptibility → preventive behavior	0.241	0.067	6.833	< 0.001
Total effects	0.411	Knowledge → preventive behavior	0.126	0.069	3.635	< 0.001

Abbreviations: C.R, critical ratio; HBM, health belief model; MSPs, musculoskeletal pains.

## 4 Discussion

The prevalence of MSPs among nurses is an important occupational health concern, often attributed to the physically demanding nature of their job [4]. The present study aimed to assessing the role of HBM constructs in MSPs prevention behaviors among nurses. This study contributes uniquely to the literature on MSPs prevention among nurses by incorporating methodological and theoretical innovations. we utilized path analysis to disentangle both direct and indirect pathways between HBM constructs and knowledge influencing preventive behaviors. This analytic approach allows a more nuanced understanding of the mechanisms underlying behavior change. Furthermore, the integration of knowledge within the HBM frameworks both a direct predictor of preventive behavior and an antecedent to perceived susceptibility, severity, and benefits responds to contemporary calls for enriching the cognitive dimension of health behavior models. Our findings highlight that knowledge contributes not only independently but also indirectly by shaping health beliefs. Importantly, this study identifies self‐efficacy as the most potent direct predictor of preventive behaviors, underscoring its role as a key target for intervention.

In the study by Afshar et al. (2019), it was reported that nurses' knowledge level was low and about 77% of nurses had MSPs. There was a significant and negative correlation between the knowledge of ergonomic principles and the ergonomic condition of the workplace with musculoskeletal injuries. They found that the knowledge of ergonomics and its application can prevent the occurrence and development of MSPs and improve the health status [35]. Felton et al. (2023) also reported that there was a significant relationship between knowledge and the use of prevention techniques. Ergonomic adjustment of equipment and postural corrections were the prevention techniques of work‐related MSPs that were often implemented [36].

According to the HBM, that perceived susceptibility to a health condition is an important factor in motivating people to take preventive measures. Regarding MSPs, the present study results confirm this notion and show that nurses who are more aware of risks and perceive themselves to be prone to MSPs are more likely to engage in preventive behaviors. In the study by Sharafkhani et al. (2016), they found that the increase in perceived susceptibility in nurses after receiving training led to an increase in back pain preventive behaviors [22].

Self‐efficacy, defined as a person's confidence in their ability to perform a behavior, has been widely recognized as an important predictor of health behavior change [37]. In the current study, self‐efficacy was found as a strong predictor of preventive behavior among nurses. This indicates that interventions aimed at strengthening nurses' belief in their ability to perform preventive measures, such as proper lifting techniques or regular exercise, could be particularly effective in reducing the incidence of MSPs. Rakhshani et al. (2024) also reported that self‐efficacy towards some barriers related to work and employer and some other things such as how to sit correctly, how to move the patient and move the wheelchair in nurses can be effective to prevent MSPs [38]. Moreover, in the study by Sari et al. (2018), they reported that back pain preventive behavior was directly and positively related to self‐efficacy [39]. Martinez‐Calderon et al. (2018) observed that high levels of self‐efficacy are associated with greater physical performance, participation in physical activity, health status, work status, performance satisfaction, and with lower levels of pain intensity, illness and depressive symptoms [40].

The constructs of the HBM, perceived severity, perceived barriers, and perceived benefits play a crucial role in understanding health behaviors. However, recent findings suggest that these constructs did not significantly predict preventive behaviors in this demographic.

Perceived severity refers to an individual's belief about the seriousness of a health issue and its consequences. In the context of musculoskeletal pain, nurses may understand the potential impact of such conditions on their health and work performance. However, despite recognizing the severity, they might not engage in preventive behaviors due to a sense of invulnerability or a belief that they can manage symptoms as they arise. Studies indicate that while awareness of severity is present, it does not always translate into action if individuals do not feel directly threatened or if they believe their current practices are sufficient to mitigate risks [41, 42].

Perceived barriers encompass the obstacles that individuals believe hinder them from adopting preventive behaviors. In healthcare settings, nurses often face significant barriers, such as time constraints, workload pressures, and lack of access to ergonomic resources. These barriers can overshadow the perceived benefits of preventive actions. For instance, even if nurses acknowledge the importance of taking breaks or using proper lifting techniques, the immediate demands of their job may prevent them from implementing these strategies. Research has shown that when perceived barriers are high, they can diminish motivation to engage in preventive behaviors [22].

Perceived benefits involve an individual's belief in the efficacy of taking specific actions to reduce risk or harm. While nurses may recognize that certain behaviors, like using ergonomic equipment or participating in training could reduce their risk of musculoskeletal pain, they might undervalue these benefits if they do not see immediate results or if they believe such measures are inconvenient. Additionally, if nurses have previously experienced pain but did not find relief through preventive measures, their confidence in these benefits may wane [42, 43].

In final, the findings of this study highlight the critical role of self‐efficacy and perceived susceptibility in motivating preventive behaviors among nurses regarding MSPs. These results underscore the importance of integrating targeted training programs focusing on enhancing nurses' self‐efficacy and risk perception. Such programs could be effectively led by physiotherapists or orthopedic specialists and incorporated within ergonomic training, physical conditioning sessions, and behavioral coaching. Integrating psychological constructs with physical interventions may contribute significantly to reducing the incidence of MSPs in nursing populations.

## 5 Limitations

This study employed a cross‐sectional design, which, although effective for identifying associations between knowledge, health beliefs, and preventive behaviors at a single time point, inherently limits the ability to infer causal relationships. Consequently, the directionality of effects among variables cannot be definitively established. Future longitudinal or interventional studies are recommended to more thoroughly investigate causal pathways and temporal changes.

Furthermore, while the statistical analyses including path analysis were conducted rigorously, some limitations related to potential biases and confounding variables should be acknowledged. The use of self‐reported questionnaires may introduce recall bias and social desirability bias, potentially affecting the accuracy of participants' responses regarding their preventive behaviors and beliefs. Additionally, certain relevant confounding factors, such as workplace ergonomic conditions, psychosocial stressors, workload, and organizational support and culture‐specific measures (e.g., power distance index, collectivism scales), were not measured and could influence both health beliefs and behaviors related to MSPs prevention. Future research should incorporate these variables and consider longitudinal designs to improve causal inference and better control for confounding influences. One other limitation of this study is the use of a binary (yes/no) measure to assess the presence of MSPs. While this approach simplified data collection and reduced respondent burden among busy nurses, it does not capture detailed aspects of pain such as intensity, duration, functional impact, or specific anatomical locations. Nevertheless, this method was appropriate for the study's focus on health beliefs and preventive behaviors. Future research could benefit from more comprehensive assessment tools to provide deeper clinical insights.

## 6 Conclusion

Building upon these findings, healthcare organizations should prioritize the development and implementation of targeted educational programs that not only raise nurses' knowledge about MSPs risks but also enhance their perceived susceptibility, thereby motivating preventive action. Such programs should be tailored to the specific work environments nurses face, incorporating hands‐on training in ergonomic principles and safe patient handling techniques.

Moreover, the significant role of self‐efficacy highlights the need for interventions that actively build nurses' confidence and skills through skill‐based workshops, simulation exercises, and peer support or mentoring systems. These approaches can foster sustainable behavioral change by empowering nurses to consistently apply preventive measures in their daily duties.

At the organizational level, creating a supportive workplace culture is crucial. This includes ensuring the availability of appropriate ergonomic equipment, setting realistic workloads, and promoting management commitment to occupational health and safety policies that reduce physical strain. Policy makers and healthcare administrators should incorporate these strategies into routine occupational health frameworks, prioritizing continuous staff training and ergonomic risk assessments. Finally, recognizing that perceived severity, barriers, and benefits act within complex cognitive, emotional, and systemic contexts, future interventions should adopt a multifaceted approach. This approach must consider not only individual beliefs but also address organizational and systemic barriers, such as staffing constraints or workplace design, that may hinder preventive behaviors. By doing so, healthcare systems can foster a comprehensive culture of musculoskeletal health promotion, ultimately reducing the burden of MSPs among nurses.

## Author Contributions

**Soudabeh Yarmohammadi** and **Esmaeil Fakharian** contributed to conceptualization, data curation, formal analysis, investigation, methodology, project administration, resources, software, supervision, validation, visualization, writing – original draft, and writing – review and editing, while **Azam Jahangiri Mehr** and **Mojtaba Sehat** contributed to formal analysis, methodology, supervision, writing – original draft, and writing – review and editing.

## Funding

The authors received no specific funding for this work.

## Conflicts of Interest

The authors declare no conflicts of interest.

## Transparency Statement

The lead author Soudabeh Yarmohammadi affirms that this manuscript is an honest, accurate, and transparent account of the study being reported; that no important aspects of the study have been omitted; and that any discrepancies from the study as planned (and, if relevant, registered) have been explained.

## Data Availability

The data supporting the findings of this study are available from the corresponding author upon reasonable request. Dr. Soudabeh Yarmohammadi had full access to all of the data in this study and takes complete responsibility for the integrity of the data and the accuracy of the data analysis.

## References

[1] H. Yarmohammadi , S. H. Niksima , S. Yarmohammadi , A. Khammar , H. Marioryad , and M. Poursadeqiyan , “Evaluating the Prevalence of Musculoskeletal Disorders in Drivers Systematic Review and Meta‐Analysis,” Journal of Health and Safety at Work 9, no. 3 (2019): 221–230.

[2] T. K. Gill , M. M. Mittinty , L. M. March , et al., “Global, Regional, and National Burden of Other Musculoskeletal Disorders, 1990–2020, and Projections to 2050: A Systematic Analysis of the Global Burden of Disease Study 2021,” Lancet Rheumatology 5, no. 11 (2023): e670–e682. 37927903 10.1016/S2665-9913(23)00232-1 PMC10620749

[3] D. Bernal , J. Campos‐Serna , A. Tobias , S. Vargas‐Prada , F. G. Benavides , and C. Serra , “Work‐Related Psychosocial Risk Factors and Musculoskeletal Disorders in Hospital Nurses and Nursing Aides: A Systematic Review and Meta‐Analysis,” International Journal of Nursing Studies 52, no. 2 (2015): 635–648, 10.1016/j.ijnurstu.2014.11.003. 25480459

[4] K. S. Krishnan , G. Raju , and O. Shawkataly , “Prevalence of Work‐Related Musculoskeletal Disorders: Psychological and Physical Risk Factors,” International Journal of Environmental Research and Public Health 18, no. 17 (2021): 9361, 10.3390/ijerph18179361. 34501950 PMC8430476

[5] J. Jacquier‐Bret and P. Gorce , “Prevalence of Body Area Work‐Related Musculoskeletal Disorders Among Healthcare Professionals: A Systematic Review,” International Journal of Environmental Research and Public Health 20, no. 1 (2023): 841, 10.3390/ijerph20010841. 36613163 PMC9819551

[6] N. M. Daraiseh , S. N. Cronin , L. S. Davis , R. L. Shell , and W. Karwowski , “Low Back Symptoms Among Hospital Nurses, Associations to Individual Factors and Pain in Multiple Body Regions,” International Journal of Industrial Ergonomics 40, no. 1 (2010): 19–24, 10.1016/j.ergon.2009.11.004.

[7] W. Sun , L. Yin , T. Zhang , H. Zhang , R. Zhang , and W. Cai , “Prevalence of Work‐Related Musculoskeletal Disorders Among Nurses: A Meta‐Analysis,” Iranian Journal of Public Health 52, no. 3 (2023): 463–475, 10.18502/ijph.v52i3.12130. 37124897 PMC10135498

[8] J. L. Flaubert , S. Le Menestrel , D. R. Williams , and M. K. Wakefield , National Academies of Sciences E ., Medicine. Supporting the Health and Professional Well‐Being of Nurses. *The Future of Nursing* 2020–2030: Charting a Path to Achieve Health Equity: National Academies Press (US); 2021, 10.17226/25982. 34524769

[9] Ł. Rypicz , P. Karniej , I. Witczak , and A. Kołcz , “Evaluation of the Occurrence of Work‐Related Musculoskeletal Pain Among Anesthesiology, Intensive Care, and Surgical Nurses: An Observational and Descriptive Study,” Nursing & Health Sciences 22, no. 4 (2020): 1056–1064, 10.1111/nhs.12767. 32767424 PMC7754151

[10] A. Koyuncu , K. Kaya , O. Kaya , and A. Yava , “The Impact of Work‐Related Musculoskeletal Pains on Routine Tasks Among Operating Room Nurses: A Multicenter Cross‐Sectional Study,” Pain Management Nursing: Official Journal of the American Society of Pain Management Nurses 26 (2025): 88, 10.1016/j.pmn.2024.08.003. 39277454

[11] K. E. Glanz , F. M. E. Lewis , and B. K. Rimer Health Behavior and Health Education: Theory, Research, and Practice: Jossey‐Bass/Wiley; 1990.

[12] K. Glanz , B. K. Rimer , and K. Viswanath Theory, Research, and Practice in Health Behavior and Health Education. 2008.

[13] M. van Ryn and C. A. Heaney , “What's the Use of Theory?,” Health Education Quarterly 19 (1992): 315–330. 1517095 10.1177/109019819201900304

[14] K. Glanz and D. B. Bishop , “The Role of Behavioral Science Theory in Development and Implementation of Public Health Interventions,” Annual Review of Public Health 31, no. 1 (2010): 399–418. 10.1146/annurev.publhealth.012809.103604 20070207

[15] E. Norozi , F. Nazari , and M. Moodi , “The Effect of Educational Intervention Based on the Health Belief Model on Osteoarthritis‐Preventive Behaviors in Middle‐Aged Women,” Journal of Education and Health Promotion 9, no. 1 (2020): 327, 10.4103/jehp.jehp_436_20. 33426131 PMC7774611

[16] S. Punlomso , P. Srimuang , and K. Tudpor , “Fall Prevention by Otago Exercise Program Based on Health Belief Model in Community‐Dwelling Older Persons,” Indian Journal of Physiotherapy & Occupational Therapy 14, no. 1 (2020): 245–252, 10.5958/0973-5674.2020.00044.1.

[17] M. Conner and P. Norman , “Predicting Health Behaviour: A Social Cognition Approach,” Predicting Health Behaviour 2 (2005): 1–27.

[18] K. Glanz , B. K. Rimer , and K. Viswanath , Health Behavior: Theory, Research, and Practice (John Wiley & Sons, 2015).

[19] S. Habibi , S. S. Tavafian , R. Maghbouli , and A. Montazeri , “The Effectiveness of Multidisciplinary Interventions Based on Health Belief Model on Musculoskeletal Pain in the Elderly Living in Nursing Homes: A Study Protocol for a Randomized Controlled Trial,” Trials 25, no. 1 (2024): 406, 10.1186/s13063-024-08243-1. 38907349 PMC11191210

[20] E. Anyfantopoulou and P. Theofilou , “Exploring Social Support, Pain Self‐Efficacy and Health Beliefs in Older Adults With Musculoskeletal Disorders,” Series of Clinical and Medical Case Reports and Reviews 1 (2023): 1–8.

[21] A. Heidari , M. Falahati , A. Coetzer‐Liversage , A. Biabani , and M. Karimy , “Prediction of Accident‐Proneness Among a Sample of Iranian Workers: Usefulness of an Adjusted Version of the Health Belief Model With Spiritual Health,” BMC Psychology 12, no. 1 (2024): 474, 10.1186/s40359-024-01956-7. 39252091 PMC11384685

[22] N. Sharafkhani , M. Khorsandi , M. Shamsi , and M. Ranjbaran , “The Effect of an Educational Intervention Program on the Adoption of Low Back Pain Preventive Behaviors in Nurses: An Application of the Health Belief Model,” Global Spine Journal 6, no. 1 (2016): 29–34, 10.1055/s-0035-1555658. 26835199 PMC4733379

[23] H. Gilasi , F. Mohebbi , and N. Sharifi , “Examining Occupational Stress and Its Related Factors in Nurses Working in the Educational Hospitals of Kashan University of Medical Sciences,” Iranian Journal of Nursing and Midwifery Research 30, no. 4 (2025): 538–543, 10.4103/ijnmr.ijnmr_372_23. 40832502 PMC12360769

[24] E. Mianehsaz , M. Tabatabaei , M. Kashani , H. Badi , and H. Rahimi , “Evaluating Musculoskeletal Disorders and Their Ergonomic Risk Factors Among Office Workers of a Large Public Hospital in Iran,” International Archives of Health Sciences 9, no. 1 (2022): 35–40, 10.4103/iahs.iahs_68_21.

[25] S. E. Bassett , Nurses' Perceptions of Occupational Self‐efficacy/workplace Autonomy Related to Organizational/structural Empowerment (Capella University, 2017).

[26] A. Dehdashti , S. Mehralizadeh , and Z. Mahjoubi , “Workplace Stresses and Musculoskeletal Disorders Among Nurses: A Cross‐Sectional Study,” Middle East Journal of Rehabilitation and Health Studies 4, no. 4 (2017): 57480, 10.5812/mejrh.57480.

[27] M. Heidari , M. Ghodusi Borujeni , P. Rezaei , and S. Kabirian Abyaneh , “Work‐Related Musculoskeletal Disorders and Their Associated Factors in Nurses: A Cross‐Sectional Study in Iran,” Malaysian Journal of Medical Sciences 26, no. 2 (2019): 122–130, 10.21315/mjms2019.26.2.13. PMC6687215 31447615

[28] R. Rashidi and R. Mohammadi , “Prevalence and Risk Factors of Musculoskeletal Disorders in Nurses Working in Khorramabad Teaching Hospitals in 2019,” Yafteh 23 (2021): 1–11.

[29] H. Taghinejad , A. Azadi , Z. Suhrabi , and M. Sayedinia , “Musculoskeletal Disorders and Their Related Risk Factors Among Iranian Nurses,” Biotechnology and Health Sciences 3, no. 1 (2016): e34473, 10.17795/bhs-34473.

[30] C. L. Peterson , K. D. Evans , and I. R. Axiotis , “Sonographer Scanning Practices and Musculoskeletal Injury: Evaluation of an Occupational Health Issue Using the Health Belief Model,” Journal of Diagnostic Medical Sonography 33, no. 5 (2017): 412–418, 10.1177/8756479317727460.

[31] S. Yang , L. Li , L. Wang , J. Zeng , B. Yan , and Y. Li , “Effectiveness of a Multidimensional Intervention Program in Improving Occupational Musculoskeletal Disorders Among Intensive Care Unit Nurses: A Cluster‐Controlled Trial With Follow‐up at 3 and 6 Months,” BMC Nursing 20, no. 1 (2021): 46, 10.1186/s12912-021-00561-y. 33743700 PMC7981926

[32] C. H. J. Pp Lawshe A Quantitative Approach to Content Validity 28, no. 4 (1975): 563–575.

[33] J. W. Creswell and J. D. Creswell , Research Design: Qualitative, Quantitative, and Mixed Methods Approaches (Sage publications, 2017).

[34] T. A. Lang and D. G. Altman , “Basic Statistical Reporting for Articles Published in Biomedical Journals: The Statistical Analyses and Methods in the Published Literature or the SAMPL Guidelines (Croatian Translation),” Bilten Hrvatskog Društva za Medicinsku Informatiku 30, no. 1 (2024): 52–61. 10.1016/j.ijnurstu.2014.09.006 25441757

[35] A. Mohammad , B. Abbas , and H. Narges , “Relationship Between Knowledge of Ergonomics and Workplace Condition With Musculoskeletal Disorders Among Nurses,” International Archives of Health Sciences 6, no. 3 (2019): 121–126, 10.4103/iahs.iahs_10_19.

[36] J. L. Felton , N. Kennedy , K. Thoirs , J. Alphonse , and A. E. Quinton , “Knowledge and Use of Work‐Related Musculoskeletal Disorder (WRMSD) Prevention Techniques in the Daily Practice of Final‐Year Australian Sonography Students: A Cross‐Sectional Study,” Sonography 10, no. 1 (2023): 3–9, 10.1002/sono.12334.

[37] A. Bandura , “Self‐Efficacy: Toward a Unifying Theory of Behavioral Change,” Psychological Review 84, no. 2 (1977): 191–215. 847061 10.1037//0033-295x.84.2.191

[38] T. Rakhshani , Z. Limouchi , H. Daneshmandi , A. Kamyab , and A. K. Jeihooni , “Investigating the Effect of Education Based on PRECEDE‐PROCEED Model on the Preventive Behaviors of Musculoskeletal Disorders in a Group of Nurses,” Frontiers in Public Health 12 (2024): 1371684, 10.3389/fpubh.2024.1371684. 38562258 PMC10982381

[39] S. A. A. Y. Sari , D. Indarto , and M. Wijaya , “Application of Health Belief Model on Preventive Behaviors of Patients With Low Back Pain,” Journal of Health Promotion and Behavior 3, no. 3 (2018): 192–198, 10.26911/thejhpb.2018.03.03.06.

[40] J. Martinez‐Calderon , C. Zamora‐Campos , S. Navarro‐Ledesma , and A. Luque‐Suarez , “The Role of Self‐Efficacy on the Prognosis of Chronic Musculoskeletal Pain: A Systematic Review,” Journal of Pain 19, no. 1 (2018): 10–34, 10.1016/j.jpain.2017.08.008. 28939015

[41] R. Bayrami , S. Masudi , A. Didarloo , and H. Nournezhad , “Determining the Predictors of Preventive Behaviors Adopted by Pregnant Women Against COVID‐19 Based on the Health Belief Model Constructs: A Cross Sectional Study,” BMC Women's Health 24, no. 1 (2024): 528, 10.1186/s12905-024-03305-7. 39304849 PMC11414123

[42] A. Alyafei and R. Easton‐Carr The Health Belief Model of Behavior Change. StatPearls 2024. 39163427

[43] M. Vaezi , “The Effect of Educational Interventions Based on Health Belief Model in Adopting Preventive Behaviors of Musculoskeletal Problems in Female Afghan Health Workers,” International Journal of Musculoskeletal Pain Prevention 1, no. 3 (2016): 137–142.

